# Carapics: A Web-based Platform for Semi-quantitative Analysis of Structural Change in Biological Samples

**DOI:** 10.17912/micropub.biology.002048

**Published:** 2026-04-06

**Authors:** Gary H. Dickinson, Sejong Yoon, Nate Sorvino, Sameer Kamal, Corin J. Hoppe, Isra Ahmad, W. Christopher Long

**Affiliations:** 1 Department of Biology, The College of New Jersey, Ewing, New Jersey, United States; 2 Department of Computer Science, The College of New Jersey, Ewing, New Jersey, United States; 3 Resource Assessment and Conservation Engineering Division, NOAA, National Marine Fisheries Service, Alaska Fisheries Science Center, Kodiak, Alaska, United States

## Abstract

Environmental stress can alter and damage biological structures with functional consequences. Inspired by observations of Tanner crabs after 2-year exposure to ocean acidification, we observed that crabs held at low pH appeared to have damaged claws. Patterns, however, were complex and three-dimensional, making quantification difficult. We developed a survey-based approach where evaluators could score the extent of damage in images of claws. We present a software tool for the creation of image surveys. The software allows users to quickly upload batches of images, parse file names, and generate surveys. Collected data is returned to the user, associated with treatment conditions.

**Figure 1. Carapics Google Apps-based biological specimen annotation management database:** **(A)** Carapics homepage. An overview and tutorial on how to upload images, create surveys, and access data is provided. Users can navigate through the app using the navigation bar within the header. **(B)** Upload images. Images are uploaded as a batch from a local drive. The app can associate treatment conditions with each image, which the user can either enter manually, or the app can parse conditions from file names if a file name template is entered. **(C)** Create form. Users can select images to be incorporated into a survey from previously uploaded images. A form title, description, and the specific question to be asked will be displayed in a Google form. Inset: example of a Google Form created with the app. **(D)** Form data. Survey responses are collated and associated with treatment conditions for each image (as entered when uploading images). Data can be exported as an Excel or .csv file.

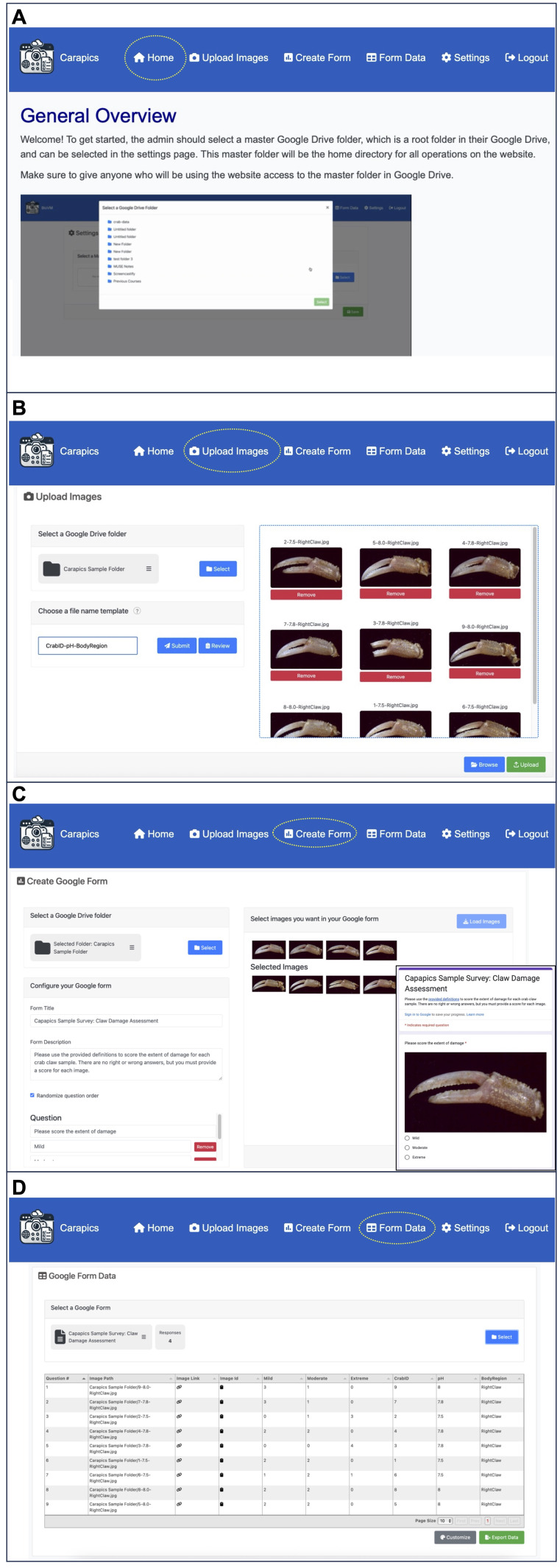



## Description

The relationship between structure and function is a central tenet of biology (Herman et al., 2021). At each level of biological complexity, structural alterations have the potential to affect functionality. This relationship is particularly relevant within the context of ongoing environmental change. For example, ocean acidification, a reduction in pH of ocean waters as a result of dissolution of atmospheric CO _2_ , can result in altered (increased or decreased) size and density/porosity of protective shells and exoskeletons, as well as dissolution or malformation of calcified structures in some taxa (reviewed by Byrne & Fitzer, 2019; Figuerola et al., 2021; Fitzer et al., 2019; Leung et al., 2022; Siegel et al., 2022). Such structural changes can alter their functionality.

A challenge in assessing the extent of structural change in a biological material, however, is identifying quantifiable metrics that accurately represent the complex, three-dimensional material as a whole. We were originally inspired by observations of exoskeleton structure in Tanner crab, *Chionoecetes bairdi,* following long-term (2-year) ocean acidification exposure (Dickinson et al., 2021). Crabs were exposed to one of three seawater pH levels (ambient: 8.1, or reduced: 7.8 or 7.5) simulating predicted ocean chemistry conditions over the next 100-200 years. A number of exoskeletal properties were quantified using standard techniques (e.g., thickness and micromechanical properties of the composite layers of the exoskeleton, elemental content, the polymorph of calcium carbonate present), but we also observed exoskeletal damage, particularly to the claws of crabs, that could not be readily quantified. For example, the dactyl and pollex (the finger-like portions of the claw) were extensively pitted and eroded, particularly in crabs held at the lowest pH level. Further, the tooth-like-denticles on these dactyls, which are more dense and mechanically resistant than other portions of the claw (Rosen et al., 2020), were worn away in many individuals.

We considered a number of options for how best to report these observations of exoskeleton damage. One option would be to simply provide representative images, perhaps with a full set of images in a supplement. Simply providing images, though, is non-quantitative and therefore precludes statistical analysis of the extent of damage. Further, choice of representative images can be subjective and is prone to bias, ignoring individual variability that is inherent to biological structures, and most readers would not review supporting materials. We could measure one or multiple structural characteristics (e.g., height or presence/absence of denticles, area of continuous dissolution), but such measurements can be logistically challenging, forcing one to make linear or two-dimensional measurements of three-dimensional damage, and may leave out more nuanced signs of damage that do not fit the definition of the metric assessed. Lastly, we could employ a three-dimensional imaging approach (e.g., μCT scanning), but such an approach is costly, requires extensive technical and computational expertise, and commercially available calibration standards for μCT scanning (phantoms) are based on vertebrate bone and not calcium carbonate (Kimoto et al., 2023).

As an alternative to these approaches, we developed a survey-based assessment of claw damage (Dickinson et al. 2021). Evaluators who had general knowledge of crustacean biology and ocean acidification, but who were not involved in the Tanner crab project, were given a series of claw images and asked to view the image holistically and provide a score for the extent of damage (mild, moderate, or extreme) based on set definitions. Images were presented blind in terms of pH exposure and in a randomized order, and a discrete score was required from the evaluator for each image. Responses from multiple independent evaluators were pooled and served as the basis for statistical assessments. Statistical tests supported our qualitative observations of damage, with a greater extent of damage in the claws of crabs exposed to reduced pH (7.8 or 7.5) as compared to those at ambient pH.

Although this approach sufficed, survey creation and deployment and response collection were cumbersome and required substantial bookkeeping to ensure that scores for blinded and randomized images were correctly coded to their respective pH treatment. Here, we present a Google Apps-based biological specimen annotation management database called Carapics (Fig. 1). The goal in developing the app was to greatly streamline the image management, survey creation and dissemination, and response collection process. The Carapics web application and survey approach was originally designed to assess the extent of damage on crustacean exoskeletons, but could be used to assess visual differences among any type of biological samples from the macro to micron scales. Carapics is compatible with multiple file formats and users can acquire images using methodology (e.g., digital camera, light microscope, electron microscope) and image capture software appropriate for their study.

To generate surveys using Carapics, image files are batch-uploaded from a local drive to a user’s Google Drive through the Carapics app ( Fig. 1B ). Carapics will automatically parse file names if formatting is consistent (e.g., sampleID-pH-temperature), or a user can manually label imported images with a sample ID and treatment conditions. To create a survey, users then select the specific images to be included, add a title and description, and add the specific question (scoring options) to be asked ( Fig. 1C ). Creating the survey generates a Google Form with an image embedded for each question ( Fig. 1C, inset). File names and treatment conditions are not displayed with images and researchers have the option of randomizing the order that images are presented. Surveys can be distributed to evaluators through a weblink, as would be done for any Google Form survey. Once evaluators have completed the survey, researchers can view scores through the Carapics app ( Fig. 1D ). Each image is displayed with the sample ID and treatment conditions initially entered, along with the number of responses for each score. Data can be downloaded from the app as an Excel or CSV file.

Once installed, use of the Carapics database alleviates many of the challenges associated with creating and disseminating image-based surveys. Researchers must carefully consider the evaluators chosen (e.g., level of expertise, previous knowledge of project), the number of evaluators to employ, if training beyond the description included in the survey is required, and appropriate statistical analyses for collected scores. Although image-based surveys may not be appropriate for all biological structures, particularly if there is a standard method in the field for quantifying that structure, the approach provides a useful tool in assessing complex three-dimensional structures. The approach is particularly amenable to outreach efforts, citizen science, and classroom (e.g., active learning) use. For example, researchers may deliver a short presentation or lead a discussion with community members or an undergraduate class, and then give the group time to complete an image survey. We strongly encourage researchers to follow-up at a subsequent class or event, presenting the findings directly to participants who have contributed; not only does this continue the outreach efforts, but it also helps participants to feel part of the full research process. We aim to expand Carapics in future iterations, including AI-driven, guided annotations of images to inform the scoring decisions of evaluators.

Code, system requirements, and installation instructions for Carapics: https://github.com/yoonsejong/carapics

A sample survey created with Carapics: https://forms.gle/D2ZtQd9zkv9RyEkR9

## Methods

The software design of the Carapics database took several practical needs of biological scientists into account. Particularly, one of the goals of Carapic’s software design was to make sure users can easily use the deployed system without extensive training. Carapics system development consists of three components: (a) web application server, (b) Python-based backend and JavaScript/HTML/CSS frontend, (c) interface with Google Cloud API.

For the web application server, users can use any web server choice. The Carapics application was developed using nginx webserver running on Ubuntu 24.04 server. However, the web server must be properly configured to use HTTPS and a domain. These are required to use Google Cloud Application Programming Interface (API) with Open Auth (OAuth) authentication. Setting up a web server satisfying the requirements requires some technical fluency, but step-by-step instructions can be easily found on the internet (a suggested tutorial can be found on the Carapics Github repository). Alternatively, users may benefit from commercial web hosting services.

Carapics’ core backend is mostly written in Python. We adopted a Python-based Flask web application framework for efficient implementation. To run Python-based web applications, one needs a web application container or web server gateway interface (WSGI) for smooth communication, e.g., request forwarding, between the web server and the Python application. For Python, a popular choice is Gunicorn. For security reasons, Gunicorn does not run as a frontend web server itself. Rather, it links with another frontend webserver, e.g., nginx or Apache. In addition, to efficiently handle multi-part, extensive file transfers that are essential for our application (e.g., image file uploads), Flask-SocketIO library was employed. This allows permanent connection between client and server, allowing responsive file upload progress tracking. For the frontend web pages, third party JavaScript libraries were adopted to implement some features. Two notable ones are Notyf and Tabulator. Notyf was adopted to display various update notifications, which are useful to track updates for various time-consuming tasks, e.g., file uploads. Tabulator excels in displaying tabular information, e.g., metadata stored in the database, or browsing survey outcomes in a curated manner.

Interfacing with Google Cloud API is a key component of Carapics. With the OAuth, users can use their personal Google account, or institutional Single Sign On (SSO) credentials if their institution uses Google Cloud. Image files are directly stored into a specific Google Drive folder of user’s choice, allowing them to efficiently manage their data with familiar user interface and storage structure. A user can use the Carapics’ web interface to input, edit, and view any metadata, that are stored as a Google Sheet. Since the entire text-based information is stored in a human-readable spreadsheet, users can easily manage the metadata directly if needed. Surveys are generated as Google Forms. Once created, users can easily browse or open particular surveys to change any settings or disseminate to annotators.

## Data Availability

Description: Carapics Version 1.0 source code. Resource Type: Software. DOI: https://doi.org/10.22002/p7m51-kb349
